# Quantitative ^1^H NMR in Pharmaceutical and Biomedical Analyses: Methodologies and Applications

**DOI:** 10.3390/molecules31122010

**Published:** 2026-06-09

**Authors:** Shangxiao An, Weiyi Zheng, Qi Tang, Guofang Shen, Yi Wang, Hua Hua, Junning Zhao, Yu Tang

**Affiliations:** 1 School of Pharmacy, Chengdu University of Traditional Chinese Medicine, Chengdu 611137, China; 2 Pharmaceutical Informatics Institute, School of Pharmacy, NMPA Center for Innovation and Research in Regulatory Science, Zhejiang University, Hangzhou 310058, China; 3 Hangzhou Institute for Food and Drug Control, Hangzhou 310022, China; 4 Sichuan Institute for Translational Chinese Medicine, Chengdu 610200, China

**Keywords:** quantitative ^1^H NMR, drug analysis, biomedical analysis, metabolomics, quality control

## Abstract

Quantitative ^1^H NMR (qNMR) is a versatile analytical tool that provides simultaneous structural and quantitative information without the need for analyte-specific standards. This review summarizes its key methodological fundamentals and broad applications in both pharmaceutical and biomedical analysis. In drug analysis, qNMR enables content determination and purity assessment of small molecules, polysaccharides and glycoconjugates, synthetic polymers, and complex herbal medicines. In biomedical analysis, it serves as a powerful platform for metabolomics profiling, real-time monitoring of cellular processes, and absolute quantification of metabolites in biofluids and tissues. Recent and emerging technological advancements, including hyperpolarization, quantum mechanical spectral analysis, artificial intelligence, and deep learning, hold great promise for further enhancing sensitivity, resolving power, and automation. With ongoing integration into pharmacopoeial standards and regulatory frameworks, qNMR is poised to expand its role in both routine quality control and cutting-edge biomedical research.

## 1. Introduction

Nuclear magnetic resonance (NMR) spectroscopy relies on a fundamental principle: atomic nuclei with a non-zero spin align when placed in an external magnetic field. When exposed to radiofrequency radiation, these nuclei absorb radiofrequency radiation at specific frequencies, causing transitions between energy levels [1,2,3]. The detected resonance signals provide a wealth of structural information, including the type, number, and chemical environment of the nuclei. NMR encompasses a range of techniques, including ^1^H, ^13^C, ^19^F, ^31^P, and various two-dimensional experiments, each offering unique advantages for qualitative and quantitative analysis.

Quantitative NMR has emerged as a powerful analytical approach due to its high repeatability and reproducibility, independence from chromophores, universal detectability across all molecules, non-destructive nature, and sample reusability. Unlike HPLC-UV, LC-MS, and GC-MS, which strictly depend on chromophores, ionization efficiency, and analyte volatility, respectively, quantitative NMR offers universal detection for any nucleus with a non-zero spin quantum number. A major advantage of quantitative NMR is that it eliminates the need for an analyte-specific reference standard; instead, any calibrated internal standard suffices, whereas chromatographic methods require identical reference materials. Furthermore, quantitative NMR simultaneously provides comprehensive structural insights, including chemical shifts, coupling constants, and multiplicities, within a single experiment, while chromatography yields only retention times or *m*/*z* ratios. Despite these advantages, quantitative NMR exhibits lower sensitivity and demands larger sample quantities. Method selection ultimately depends on the specific analytical objective: quantitative NMR excels at reference material certification and complex mixture fingerprinting, whereas LC-UV and LC-MS methods remain preferred for trace analysis.

Quantitative NMR is nearly as old as NMR itself. Following the discovery of NMR in 1946 by Purcell, Bloch, and colleagues, the quantitative potential was recognized in the early 1960s. However, early textbook literature often underestimated qNMR’s precision, with some sources estimating integration errors of 5–10% [4]. A pivotal methodological advance came with the introduction of Fourier-transform NMR by Ernst and Anderson in 1966, which dramatically improved sensitivity and enabled practical quantitative measurements [5]. Since the early 2000s, the work of Pauli and colleagues has systematically established quantitative ^1^H NMR (qNMR) as a primary ratio method for natural product reference compound validation [6,7,8]. Owing to the high natural abundance of ^1^H (99.985%) and the ubiquitous presence of hydrogen in organic and biological molecules, qNMR offers both high sensitivity and broad applicability [9,10,11,12].

In recent decades, qNMR has evolved from a specialized technique into a routine analytical tool in both pharmaceutical and biomedical research. Its unique ability to simultaneously deliver structural elucidation and absolute quantification without requiring analyte-specific reference standards, makes it particularly attractive for applications ranging from early-stage drug discovery to clinical metabolomics. However, despite its growing adoption, challenges remain in spectral overlap, sensitivity, and standardization, which have spurred numerous methodological innovations [1]. Previous excellent qNMR reviews are now over a decade old [6,7,8]. In addition, prior reviews focused mainly on natural products, whereas this review uniquely integrates both pharmaceutical applications (small molecules, polymers, and herbal medicines) and biomedical applications (metabolomics and clinical diagnostics), providing a unified resource across disciplines.

This review provides a comprehensive overview of the current state of qNMR. Section 2 outlines the methodological fundamentals, including quantitative principles, calibration strategies, and optimization of experimental parameters. Section 3 is divided into two main parts: Section 3.1 focuses on pharmaceutical applications, covering small-molecule drugs, saccharides and polymers, and herbal medicines; Section 3.2 addresses biomedical applications, including metabolomics and disease biomarker discovery, as well as quantitative metabolite profiling in biofluids and tissues. Finally, Section 4 discusses future perspectives, with emphasis on emerging technologies, artificial intelligence, and the integration of qNMR into regulatory and pharmacopoeial frameworks.

## 2. Methodological Fundamentals of qNMR

### 2.1. Quantitative Basis and Underlying Principle

The core premise of qNMR relies on direct proportionality. Under optimized experimental conditions, the integrated area of a ^1^H NMR signal directly reflects the number of protons giving rise to that resonance [6,7,8]. This relationship renders NMR a primary ratio method for quantitative analysis. For different signals within the same spectrum, the ratio of signal integrals directly reflects the ratio of proton numbers, which can then be used to calculate molar ratios and mass fractions of analytes [13,14,15]. It is important to note, however, that this proportionality is not unconditional. It holds strictly only when the NMR experiment is performed under fully relaxed conditions. The critical parameter here is the interpulse delay (D1), which is the time allowed for nuclear spins to return to thermal equilibrium after each excitation pulse. Basically, an interpulse delay of 60 s is sufficient [8].

### 2.2. Relative Versus Absolute Quantification

Depending on the calibration strategy, qNMR can be broadly classified into relative and absolute quantification approaches [8]. Relative quantification operates without an internal calibrant. Instead, all detectable NMR signals are normalized to their one-proton equivalents. The sum of these normalized integrals is taken as “100%”. The purity of each component is then calculated as its normalized integral divided by this total sum. This method eliminates weighing errors and is particularly valuable for precious or mass-limited samples. However, it cannot account for NMR-invisible species such as inorganic salts, requires assignment of all signals and tends to overestimate purity. Absolute quantification requires the addition of a known amount of a certified internal calibrant (e.g., maleic acid, fumaric acid, or 3,5-dinitrobenzoic acid) to the sample (see Table 1 for details of commonly used internal calibrants). By comparing the integral ratio, proton number, molecular weight, and mass of the analyte and the calibrant, the absolute purity or content of the analyte can be determined [16,17,18]. This method meets GLP/GMP requirements for traceable and highly accurate purity assignment. Several practical challenges must be addressed when selecting an internal calibrant. Most importantly, the calibrant signals must not overlap with those of the analyte. Furthermore, chemical interactions may occur between the calibrant and the analyte through mechanisms such as hydrogen bonding or π-π stacking. Finally, sample recovery is impossible once the calibrant is introduced, and the use of volatile calibrants introduces the risk of analyte evaporation during the process. While internal and external standard addition protocols remain the gold standard, alternative calibration strategies include ERETIC and PULCON [19]. ERETIC generates an artificial electronic reference signal directly from the spectrometer console to serve as an in-spectrum standard, while PULCON uses the principle of reciprocity to correlate absolute signal intensities between separate sample and reference spectra based on their respective 90° or 360° pulse lengths. While these two strategies are highly valuable for specific applications, their reliance on specialized parameters and complex instrument factors prevents them from replacing standard single-tube internal calibration in mainstream qNMR workflows.

### 2.3. Establishment of Quantitative Conditions and Parameter Optimization

**Figure 1:**
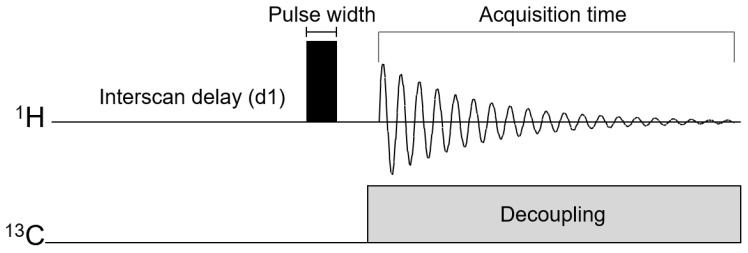
Inverse-gated decoupling pulse sequence for qNMR experiments.

The most widely used pulse sequence for qNMR is the single-pulse sequence [18]. Its basic structure consists of a D1 followed by a radiofrequency pulse (typically 30° or 90°) and acquisition of the free induction decay (FID). The relaxation delay ensures that the nuclear spins return to thermal equilibrium before the next excitation, which is essential for accurate quantification. Therefore, accurate and precise qNMR measurements require strict control of the following experimental parameters: (1) D1 and flip angle: To ensure full longitudinal relaxation of all proton signals of interest, the D1 should be at least 5–7 times the longest longitudinal relaxation time (T_1_) [16,17,20]. For a 90° flip angle, D1 = 5–7 × T_1_ is recommended; for a 30° flip angle, a shorter D1 may be used. The T_1_ values of relevant protons must be experimentally determined to set D1 appropriately. The most common method is the inversion-recovery experiment, in which a 180° pulse inverts the longitudinal magnetization, followed by a variable delay during which the magnetization recovers, and then a 90° pulse samples the remaining magnetization [6]. By measuring signal intensities as a function of the variable delay and fitting them to an exponential recovery curve, the T_1_ value for each proton signal can be accurately determined. It is critical to measure T_1_ for all protons of interest, including those of the target analyte(s) and the internal calibrant. The D1 must then be set based on the longest T_1_ value among all protons to be quantified to ensure complete relaxation of every relevant spin. (2) Signal-to-noise ratio (S/N): S/N directly determines the precision of qNMR measurements. To achieve a relative uncertainty of 1%, the quantitative peak should have an S/N ≥ 150 [8]. Crucially, this threshold must be calculated using a standardized root-mean-square (RMS) noise region rather than proprietary automated vendor scripts, ensuring cross-platform validation and reproducibility across different spectrometer software suites. (3) Data processing: Phase and baseline corrections should be performed carefully (either manually or automatically), and ^13^C satellite peaks should be excluded from the integration region. Inverse-gated decoupling (^1^H{^13^C}) is therefore recommended for qNMR experiments (Figure 1), as it collapses the ^13^C satellites into the parent resonance. In contrast to a manual integration strategy that excludes satellite signals, this approach integrates the collapsed satellites as part of the main peak, thereby improving sensitivity without sacrificing quantitative accuracy. The integration width is recommended to be 25–64 times the full width at half maximum of the peak [13,14,15]. Finally, to assist researchers in implementing qNMR methods, Table 2 summarizes recommended acquisition and processing parameters. These settings are compiled from the methodological guidelines established in the seminal qNMR literature [4,6,7,8] and are intended to serve as a practical starting point for routine quantitative analysis.

### 2.4. Influence of Magnetic Field Strength on qNMR Performance

The selection of magnetic field strength dictates the critical trade-offs between spectral resolution, sensitivity, acquisition time, and cost. Mid-field spectrometers (400–600 MHz) offer the optimal balance for routine qNMR applications, providing sufficient dispersion and sensitivity for most organic mixtures while minimizing maintenance costs [8]. Conversely, ultra-high-field systems (800–900 MHz) are essential for complex mixtures, such as untargeted metabolomics, where superior S/N are required, despite the higher capital investment [16]. Meanwhile, 60–90 MHz benchtop instruments are increasingly used for routine quality control, offering cost-effective and accessible analysis for simpler samples [21].

### 2.5. Influence of Solvent Selection on Quantification Accuracy

The choice of deuterated solvent is a critical variable that directly impacts qNMR accuracy through multiple orthogonal pathways [22,23]. First, the solvent must guarantee complete solubility of both the analyte and the calibrant to ensure true molar proportionality while remaining chemically inert to prevent degrading interactions. Second, the residual proton signals of the solvent must not overlap with the target integration zones of interest. Finally, the physical properties of the solvent profoundly alter nuclear relaxation mechanics. High-viscosity solvents, such as DMSO-*d*_6_, increase molecular rotational correlation times, which accelerates dipole–dipole relaxation and yields shorter T_1_ values. Conversely, low-viscosity solvents like CDCl_3_ prolong T_1_ relaxation times, subsequently requiring significantly longer relaxation delays to prevent signal saturation and underquantification.

## 3. Applications in Pharmaceutical and Biomedical Analysis

The versatility of qNMR extends across both pharmaceutical and biomedical fields, offering a unique combination of structural elucidation and absolute quantification without requiring analyte-specific reference standards. In pharmaceutical analysis, its applications span the entire drug development lifecycle, from impurity profiling of small-molecule active pharmaceutical ingredients to the characterization of complex biologics such as glycoconjugate vaccines and biodegradable drug delivery systems. In biomedical analysis, qNMR serves as a powerful platform for metabolomics and real-time monitoring, enabling the simultaneous identification and quantification of numerous metabolites in biofluids, tissues, and even living cells. By offering a non-destructive, rapid, and information-rich analytical profile, qNMR provides a unified approach for both quality control and mechanistic studies, solidifying its role as a cornerstone methodology in modern pharmaceutical and biomedical research.

### 3.1. Pharmaceutical Applications

#### 3.1.1. Small-Molecule Active Pharmaceutical Ingredients

**Figure 2:**
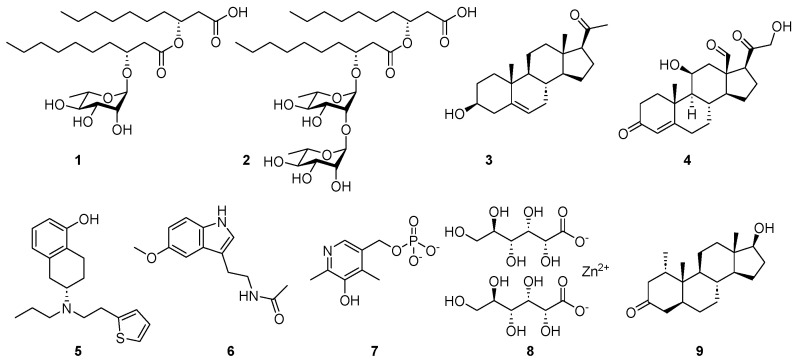
Chemical structures of representative small molecules analyzed by qNMR.

The determination of small-molecule drugs represents one of the most mature applications of qNMR, particularly when reference standards are unavailable or sample matrices are complex. Li et al. [24] developed a ^1^H NMR method for the direct quantification of mono- and di-rhamnolipids (**1** and **2** in Figure 2) without requiring high-purity standards. Using sodium p-hydroxybenzoate as an internal standard in a DMSO-*d*_6_/CD_3_OD mixed solvent, the method achieved excellent reproducibility (RSD < 2%) and accuracy (relative error < 5%). Schleiff et al. [25] validated a qNMR method for pregnenolone (**3**) in bulk substances and dietary supplements, using dimethyl sulfone as the internal standard. The method showed good linearity (0.032–3.2 mg/mL) and was successfully applied to seven bulk samples and five commercial products. Singh et al. [26] employed qNMR to determine the absolute content of aldosterone (**4**) and to identify its tautomeric forms in solution, revealing significant impurities in one commercial source that would critically affect its use as a primary calibrator. Zhou et al. [27] developed a solvent-suppressed ^1^H NMR method for the simultaneous quantification of rotigotine (**5**), mannitol, and stearic acid in prolonged-release microspheres. Using the noesyppr1d pulse sequence to suppress the water peak, the method achieved excellent recovery (98–102%) and was more efficient than traditional HPLC. Gao et al. [28] reported a qNMR method for the simultaneous determination of melatonin (**6**) and vitamin B6 (**7**) in commercial products. Using maleic acid as the internal standard in DMSO-*d*_6_, the method showed good agreement with HPLC results. Hassan et al. [29] quantified zinc gluconate (**8**) in the presence of vitamin C using maleic acid in D_2_O, validating the method for linearity, precision, and accuracy. Balayssac et al. [30] applied both quantitative ^1^H and ^13^C NMR, combined with chemometric analysis, to characterize anabolic steroid formulations, such as mesterolone (**9**), seized by law enforcement. While quantitative ^1^H NMR was sufficient for routine identification and quantification in this study, the authors found that quantitative ^13^C NMR was indispensable for discriminating between two closely related steroid structures possessing nearly identical ^1^H NMR chemical shifts. This work highlights the complementary roles of ^1^H NMR for routine quantification and ^13^C NMR for enhanced structural discrimination in complex illicit drug samples. Collectively, these studies demonstrate that qNMR is a powerful and versatile tool for small-molecule drug analysis, capable of addressing challenges ranging from the lack of reference standards to complex formulation matrices, and can be further extended by heteronuclear NMR and chemometric approaches for forensic and regulatory applications.

#### 3.1.2. Saccharides, Glycoconjugates and Synthetic Polymers

**Figure 3:**
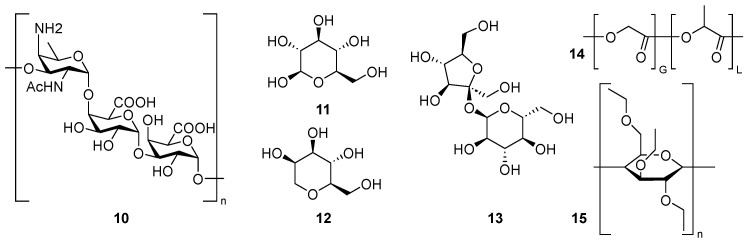
Chemical structures of representative saccharides and polymers analyzed by qNMR.

Complex saccharides and biodegradable polymers pose significant analytical challenges that qNMR is uniquely positioned to address, as it delivers both structural and quantitative information simultaneously. In a vaccine development study, Humpierre et al. [31] employed qNMR to analyze eight novel bivalent glycoconjugates. These targets were assembled from *Streptococcus pneumoniae* and *Neisseria meningitidis* capsular polysaccharides (see **10** in Figure 3 for details of the repetitive units of the selected polysaccharides). Using TSP-*d*_4_ as an internal standard in D_2_O, the characteristic anomeric and *N*-acetyl proton signals of each polysaccharide were well resolved, enabling the determination of individual polysaccharide content, the ratio between the two saccharides, and the polysaccharide-to-protein ratio. Biswas et al. [32] developed a qNMR method for the simultaneous quantification of glucose (**11**, beta form), fructose (**12**, beta form), and sucrose (**13**) in natural sweeteners (honey, jaggery, date syrup, and coconut sugar) using DSS as an internal standard. The method was fully validated with excellent linearity (R^2^ > 0.999) and accuracy, and cross-validation with UPLC showed good agreement. Qin et al. [33] developed a quantitative ^1^H and ^31^P NMR method using a single internal standard (hexamethylphosphoramide, HMPA) to simultaneously determine ribose and phosphorus contents in *Haemophilus influenzae* type b capsular polysaccharide, a key antigen for conjugate vaccines. The optimal polysaccharide concentration (15–20 mg/mL) was identified to mitigate viscosity effects, and the results aligned well with traditional colorimetric assays. In the field of polymer excipients, Wang et al. [34] utilized qNMR to monitor the erosion of biodegradable PLA/PLGA polymers in brimonidine intravitreal implants (**14**). Using ethylene carbonate as an internal standard in DMSO-*d*_6_, the method quantified the remaining polymer mass, the percentages of lactic acid and glycolic acid units, as well as trace oligomeric degradants retained in the implants. This approach offered superior specificity over gravimetric analysis and could quantify oligomers below the molecular weight cutoff of GPC. Zhao et al. [35] applied ^1^H NMR to analyze extracts from polypropylene granules, identifying catalyst residues (phthalate esters and silanes) and low-molecular-weight propylene oligomers. A secondary extraction with ethanol and *n*-hexane enabled the efficient removal of both metal-containing species and oligomers. Zhang et al. [36] established a relative qNMR method for determining ethoxy content in ethylcellulose (**15**) using a CDCl_3_/TFA-*d* solvent mixture. By integrating methyl (*δ* 1.15 ppm) and methylene/methine (*δ* 2.5–5.5 ppm) proton signals without any internal calibrant, the method achieved robust performance and was successfully validated through a multi-laboratory round robin test. These studies collectively demonstrate that qNMR is a powerful tool for the quality control of complex saccharides and polymers, from vaccines and natural sweeteners to biodegradable implants and pharmaceutical excipients.

#### 3.1.3. Herbal Medicines and Natural Products

**Figure 4:**
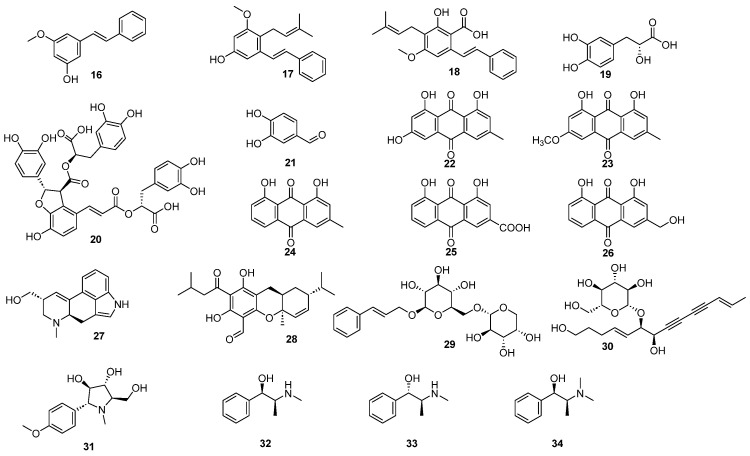
Chemical structures of representative natural products from herbal medicines.

qNMR has emerged as a powerful tool for the quality control of herbal medicines and natural products, enabling simultaneous identification and quantification of multiple bioactive constituents in complex matrices. For quality evaluation of pigeonpea (*Cajanus cajan* (L.) Millsp.), Qiu et al. [37] integrated preparative HPLC with qNMR to establish a quantitative analysis of multi-components by single marker (QAMS) method. Three key stilbenes (pinosylvin monomethylether (**16**), longistylin C (**17**), and cajaninstilbene acid (**18**) in Figure 4) were isolated and absolutely quantified by qNMR using 1,3,5-trimethoxybenzene as an internal standard, with the singlet peak of aromatic protons as the target signal for integration. The relative correction factors against resveratrol were determined, enabling simultaneous quantification without commercial standards. The method successfully revealed that leaves contained the highest total stilbene content (1.96%). Zhang et al. [38] proposed an integrated NMR strategy for elucidating the structure of a neutral homopolysaccharide (APS-N) from *Astragalus membranaceus*. Using 1D DREAMTIME TOCSY to resolve severely overlapped signals, eight glucose residues with diverse glycosidic linkage patterns were identified via empirical database matching, and their stoichiometric ratios were determined by qNMR [39]. Yan et al. [40] developed a ^1^H NMR method combined with chemometrics for the quality control of compound Danshen extract (intermediate of compound Danshen Dripping Pills). Twenty metabolites were identified, and three phenolic acids (danshensu (**19**), salvianolic acid B (**20**), and protocatechuic aldehyde (**21**)) were simultaneously quantified using TSP as an internal standard. The quantitative results showed no significant difference from UPLC, and the chemometric model successfully distinguished authentic samples from fakes. Dong et al. [41] developed a rapid qNMR method for the simultaneous determination of five bioactive free anthraquinones (emodin (**22**), physcion (**23**), chrysophanol (**24**), rhein (**25**), and aloe-emodin (**26**)) in Radix et Rhizoma Rhei. Using acetone-*d*_6_ as the solvent and 1,4-dioxane as the internal standard, the method was validated for specificity, precision, and stability, presenting a feasible alternative to HPLC. Bandla et al. [42] developed and validated a qNMR method for lysergol (**27**), an ergot alkaloid, using 1,3,5-trimethoxybenzene as an internal standard in DMSO-*d*_6_. The method demonstrated excellent specificity, precision (RSD < 2%), accuracy (96.6–100.6%), and linearity (r > 0.999), and was successfully applied to both bulk drug substances and plant extracts. Pathania et al. [43] prepared a phloroglucinol-rich fraction from *Eucalyptus camaldulensis* leaves and quantified nine phloroglucinol derivatives using qNMR with 2-ethylphenol as an internal standard. Characteristic proton signals arising from the isoprene side chains were selected and confirmed by HSQC, enabling reliable quantification without complete isolation of individual compounds, such as euglobal IIc (**28**). Xie et al. [44] established a qNMR method for Qishen Yiqi Dripping Pills, identifying 26 metabolites and simultaneously quantifying five major constituents (e.g., danshensu, rosmarinic acid) using TSP in methanol-*d*_4_. The results showed good agreement with HPLC. Tang et al. [16] developed an off-line tandem high-speed countercurrent chromatography (HSCCC) qNMR workflow for the gravimetric analysis of the *Rhodiola rosea* metabolome. By using countercurrent separation to quantitatively remove interfering proanthocyanidins, which otherwise distort both NMR and UHPLC baselines, they achieved absolute quantification of 27 metabolites across ten compound classes, for instance rosavin (**29**), with a single nonidentical internal calibrant. Tang et al. [45] employed PCA-assisted qNMR profiling to characterize natural, in vitro cultured, and artificial Calculus bovis. Species-specific markers were identified: near-negligible taurine in natural samples, choline in cultured samples, and hyodeoxycholic acid for artificial samples. The method further enabled detection and absolute quantification of adulterants (sucrose and glucose) in commercial samples. Wang et al. [17] developed an integrated strategy termed BAMLOC (biogravimetric analysis and machine learning-driven origin classification) for systematic studies of botanical materials using Codonopsis Radix as a model. Absolute qNMR quantification combined with zebrafish model-based activity index calculation identified polyacetylenes and pyrrolidine alkaloids (**30** and **31**) as the bioactive substance groups responsible for immune restoration effects. Tang et al. [21] established quantum mechanical-based digital reference materials for ephedrine-type alkaloids (ephedrine (**32**), pseudoephedrine (**33**), and methylephedrine (**34**)). By generating field-independent spin parameters (*δ* and *J*), the digital reference materials (dRMs) enabled qualitative and quantitative analysis of *Ephedra sinica* extracts and a complex traditional Chinese medicine formulation without physical reference standards, with results consistent with HPLC-UV validation. Collectively, these studies demonstrate that qNMR is a versatile and reliable platform for the quality control of herbal medicines and natural products, ranging from simple quantitation and chemometric classification to integrated qualitative–quantitative analysis, structural elucidation of polysaccharides, adulterant detection, gravimetric metabolome analysis, and the emerging concept of digital reference materials.

### 3.2. Biomedical Applications

#### 3.2.1. Metabolomics and Disease Biomarker Discovery

**Figure 5:**
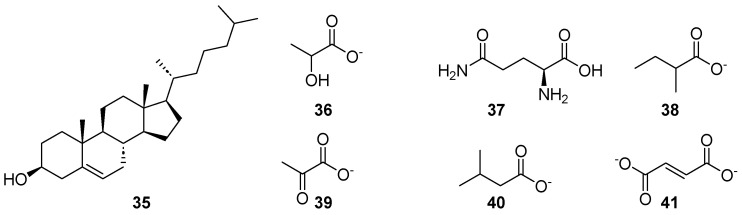
Chemical structures of representative metabolites and endogenous compounds in biomedical applications.

qNMR has also become a cornerstone platform for metabolomics and real-time monitoring in complex biological systems, offering simultaneous identification and quantification of numerous metabolites without separation. For lipoprotein profiling, Zhu et al. [46] cross-validated the NMR-based B.I.LISA method against standard enzymatic assays for cholesterol (**35**) as shown in Figure 5, triglycerides, and HDL-cholesterol in 620 plasma samples from the OMNI-Heart dietary intervention study. Despite a median deviation of −15% for triglycerides, the two methods showed high correlation (R = 0.85–0.92) and delivered consistent biological interpretations of dietary outcomes, demonstrating the comparability of NMR-based lipoprotein profiling with enzymatic methods in the context of a dietary intervention study. Hövener et al. [47] developed a low-field SABRE (Signal Amplification by Reversible Exchange) hyperpolarizer for in situ quantification of hyperpolarization, achieving a ^1^H polarization level of ≈10^−2^ in biocompatible ethanol–water mixtures, a major step toward future in vivo molecular imaging. In antibiotic research, Hoerr et al. [48] employed ^1^H NMR-based metabolomics to characterize the mechanism of action of various antibiotics by analyzing both intracellular metabolic fingerprints and extracellular metabolic footprints of *E. coli* cultures. Antibiotics targeting intracellular mechanisms produced class-specific fingerprints. In contrast, cell-wall inhibitors were distinctly categorized by their footprints, enabling correct prediction of the mode of action for untested antibiotics. Elmi et al. [49] performed the first comprehensive ^1^H NMR metabolomic characterization of porcine vitreous humor under physiological and photoreceptor degeneration conditions induced by iodoacetic acid, identifying and quantifying 40 metabolites. The observed changes (decreased lactate (**36**), increased glucose and glutamine (**37**)) validated the expected metabolic impairment, providing a reference for future ophthalmic studies. Barbieri and Luchinat [50] developed a high-cell density NMR bioreactor coupled with real-time quantitative in-cell NMR to monitor protein–ligand interactions directly in living human cells, observing the binding of drugs (acetazolamide and methazolamide) to intracellular carbonic anhydrase II and extracting concentration profiles and kinetics for free and bound states. De Graaf and Behar [51] utilized diffusion-sensitized ^1^H NMR, which uses magnetic field gradients to systematically decrease the signal intensity of molecules based on how fast they physically move (translate) through a solution, to separate low-molecular-weight metabolites from macromolecules in blood plasma based on differences in translational diffusion coefficients, enabling absolute quantification without ultrafiltration. Berezhnoy et al. [52] applied quantitative NMR-based lipoprotein analysis to serum samples from Alzheimer’s disease patients, identifying elevated HDL-4 and triglycerides in dementia groups, with gender-specific alterations suggesting HDL-4 as a potential diagnostic marker. Collectively, these studies highlight the expanding role of qNMR in biomedical analysis, from large-scale metabolomics and real-time cellular monitoring to portable diagnostic devices and multi-platform molecular characterization.

#### 3.2.2. Quantitative Metabolite Profiling in Biofluids and Tissues

The absolute quantification of small-molecule metabolites in diverse biological matrices, ranging from biofluids to tissue extracts, has been greatly facilitated by qNMR, which can be adapted for medium- to-high throughput studies. Huang et al. [53] expanded the coverage of serum quantitative metabolomics to 352 analytes (37 low-molecular-weight metabolites and 296 lipoprotein subfractions) and assessed their long-term reproducibility over 950 days, finding that 329 analytes showed CV < 20%. Application to hepatitis B virus-infected patients revealed significantly altered levels of *N*-acetylgly-coproteins, unsaturated fatty acids, and multiple lipoprotein subfractions. Guo et al. [54] applied ^1^H NMR-based quantitative metabolomics to plasma, urine, and liver extracts from hamsters fed a high-fat, high-cholesterol diet to characterize the time-dependent metabolic progression of atherosclerosis. Forty plasma, 80 urine, and 60 liver hydrophilic extract metabolites were quantified, revealing perturbations in energy homeostasis, gut microbiota functions, inflammation, and oxidative stress coupled with cholesterol and fatty acid metabolism. Shi et al. [55] developed a high-throughput qNMR method for CHO cell culture monitoring using a short relaxation delay. T_1_ values for the key metabolites in the cell culture medium were experimentally measured. Based on these, a short D1 of 4 s was used, and a correction factor, k, was calculated for each metabolite to mathematically compensate for incomplete relaxation during quantification. Twenty-seven metabolites, including 2-methylbutyrate (**38**), pyruvate (**39**), isovalerate (**40**), and fumarate (**41**), were quantified via peak fitting and multivariate curve resolution-alternating least squares (MCR-ALS), and the method successfully identified glucose, aspartate, and isoleucine as key metabolites distinguishing 500 L from 1500 L bioreactor performance. Ou et al. [56] performed a phenome-wide association study in 302 healthy Japanese individuals using qNMR. They identified 907 significant associations among 18 metabolites, 111 lipoproteins and 34 phenotypes. This work confirmed known associations, such as trimethylamine-*N*-oxide (TMAO) with cholesterol and branched-chain amino acids with BMI, and revealed distinct association patterns for HDL-1 and LDL-4 subclasses with obesity-related traits. These studies collectively demonstrate that qNMR is a versatile and robust tool for metabolite profiling across a wide range of biological samples and applications, from disease biomarker discovery to bioprocess monitoring and natural product quality control.

#### 3.2.3. Challenges and Best Practices for qNMR in Biomedical Metabolomics

Quantifying metabolites in biofluids or tissues using qNMR introduces severe challenges driven by high biological variability and matrix complexity [57]. Innate donor variations alter baseline metabolite profiles, necessitating stringent standardized sample-handling protocols to suppress confounding variables and enzymatic degradation. Spectroscopically, complex matrices generate severe signal crowding, where low-abundance biomarkers are routinely obscured by dominant water, lipid, or protein signals [57,58]. To overcome this spectral overlap, specialized acquisition pulse sequences are deployed; for example, the Carr–Purcell–Meiboom–Gill loop filters out broad macromolecular backgrounds, while 1D nuclear Overhauser effect spectroscopy achieves robust water suppression [59]. Furthermore, variations in sample pH and ionic strength shift chemical resonances and alter T_1_ relaxation behaviors [60,61]. Best practices require balancing all samples with high-capacity phosphate buffers and adding internal references like TSP or DSS to lock chemical shifts. Finally, resolving unavoidable multi-peak overlap during data analysis demands advanced post-processing deconvolution algorithms, such as targeted profiling or Bayesian line-shape fitting [60,62], to ensure baseline resolution and guarantee absolute quantification accuracy.

## 4. Conclusions and Perspectives

In conclusion, qNMR has solidified its position as a widely used and versatile analytical technique in both pharmaceutical and biomedical fields. Its principal strength lies in the unique synergy of quantitative rigor and rich structural insight within a single, non-destructive experiment. Unlike chromatographic methods that require separate runs for different analyte classes and rely on structure-dependent detector responses, qNMR offers universal detection with a uniform response factor across all proton-bearing species. This intrinsic property is firmly grounded in NMR theory. Because of it, qNMR serves as a primary ratio method capable of absolute quantification without analyte-specific standards. As evidenced throughout this review, qNMR delivers robust solutions for a wide spectrum of challenges. In pharmaceutical analysis, it enables the purity assessment of small-molecule drugs, the compositional analysis of complex biologics such as glycoconjugate vaccines, the quality control of herbal medicines, and the characterization of synthetic polymers and pharmaceutical excipients. In biomedical analysis, it serves as a powerful platform for metabolomics profiling, real-time monitoring of cellular processes, and the absolute quantification of metabolites in biofluids and tissues. The ability to extract both qualitative and quantitative information from a single experiment, combined with the non-destructive nature of NMR, makes qNMR uniquely suited for applications ranging from early-stage drug discovery to clinical diagnostics and industrial quality control.

Despite its strengths, qNMR has inherent limitations. (a) Sensitivity: compared to LC-MS (pg–fg detection), HNMR requires μmol quantities (μg–mg). (b) Spectral overlap: in complex mixtures (herbal extracts, biofluids), severe signal crowding in the aliphatic (*δ* 0.5–3.0) and carbohydrate (*δ* 3.0–4.5) regions compromises quantification. (c) NMR-invisible species: compounds lacking non-exchangeable protons (inorganic salts, silica gel residues) escape detection. (d) Dynamic range: simultaneous quantification of major components (>95%) and trace impurities (<0.1%) remains challenging, though ^13^C satellites (0.55% intensity each) serve as a useful internal threshold. (e) Expertise and cost: high-field instruments require significant capital and specialized expertise, though benchtop systems are lowering this barrier. Looking forward, several technological frontiers promise to further expand the capabilities and accessibility of qNMR. First, artificial intelligence and machine learning are poised to transform qNMR data processing and interpretation. Traditional spectral analysis relies heavily on manual phase correction, baseline correction, and peak integration, which are time-consuming and subject to operator bias. Deep learning frameworks are now emerging that can automatically identify and quantify components directly from highly overlapped ^1^H NMR spectra, even in complex mixtures where conventional integration fails [63]. Beyond quantification, AI-assisted spectral deconvolution and peak alignment are expected to dramatically improve throughput in large-scale metabolomics studies and industrial quality control settings. For instance, DEEP picker utilizes multi-layered architectures trained on large synthetic datasets to achieve expert-level peak picking and parameter estimation in highly overlapped or low-S/N regions [64]. As training databases expand and algorithms mature, fully automated, end-to-end qNMR analysis may become routine. Second, quantum mechanical spectral analysis is re-establishing itself as the gold standard for both qualitative and quantitative interpretation. Approaches such as HiFSA (^1^H iterative Full Spin Analysis) extract complete sets of chemical shifts and coupling constants directly from experimental spectra, capturing the full spin information of a molecule rather than reducing it to isolated peak integrals [20,21]. This approach reduces subjectivity, enhances reproducibility, and provides a digital “genotype” of the analyte that is independent of magnetic field strength [65]. QM-based qNMR is particularly powerful for resolving severely overlapped signals, quantifying minor components in complex mixtures, and enabling the emerging concept of digital reference materials, where physical standards are replaced by fully characterized spin parameter profiles that can be shared and reused across laboratories and instruments [65]. This digital paradigm not only addresses the limited availability of authentic reference materials for rare or regulated compounds but also aligns with the principles of open science and fair data management. Third, hyperpolarization techniques, particularly dissolution dynamic nuclear polarization (DNP), are overcoming the inherent sensitivity limitations of NMR [66]. By transiently enhancing nuclear spin polarization by several orders of magnitude, dissolution DNP enables the detection of low-abundance metabolites and even real-time monitoring of metabolic fluxes at natural isotopic abundance. While historically confined to specialized facilities, advances in polarizer automation and cryogenic technology are making DNP more accessible. When combined with fast acquisition methods [67], DNP-enhanced qNMR holds promise for extending quantitative analysis to sample-limited or low-concentration scenarios, such as single-cell metabolomics, clinical biopsy analysis, and pharmacokinetic studies. Other hyperpolarization techniques, such as SABRE (Signal Amplification by Reversible Exchange) [46], offer complementary advantages in terms of experimental simplicity and compatibility with aqueous, biocompatible solvents, further broadening the scope of in vivo and real-time applications. Fourth, benchtop NMR spectrometers (low-field, permanent-magnet systems) are democratizing access to qNMR [68]. Historically, high-field superconducting magnets (400 MHz and above) have been considered necessary for resolving complex mixtures. However, recent generations of benchtop instruments (40–90 MHz for ^1^H) offer dramatically lower cost, minimal siting requirements, and simplified operation [21,69], making qNMR deployable in industrial process lines, field laboratories, and educational settings. Low-field benchtop NMR systems suffer from severely compressed chemical shift dispersion, causing significant signal crowding and complex peak overlap. This resolution loss often yields second-order coupling patterns that hinder straightforward baseline integration [70]. Consequently, benchtop qNMR quantification is strictly limited to isolated target resonances or simplified, low-complexity mixtures. Although lower field strength exacerbates spectral overlap, this limitation can be mitigated by advanced pulse sequences (e.g., pure-shift methods and selective excitation) and by transferring spin parameter knowledge from high-field reference data via QMSA. Fifth, the integration of qNMR into manufacturing and regulatory frameworks is accelerating. In the pharmaceutical industry, qNMR is increasingly recognized as a powerful process analytical technology (PAT) tool for real-time monitoring of chemical and biotechnological processes [55,71]. Its ability to simultaneously quantify multiple components without separation makes it particularly attractive for monitoring bioreactor nutrients and metabolites, verifying raw material quality at point of receipt, and enabling in-line or at-line control of drug substance and drug product manufacturing.

Simultaneously, regulatory acceptance of qNMR is growing. Major pharmacopeias, including the United States Pharmacopeia and the Chinese Pharmacopoeia, have incorporated qNMR methods for purity assessment, reference standard characterization, and excipient testing. The revision of USP General Chapters <761> and <1761> on NMR spectroscopy reflects a concerted effort to standardize terminology, establish best practices, and provide regulatory guidance for both qualitative and quantitative NMR applications [72]. Furthermore, the development of certified reference materials specifically designed for qNMR (e.g., NIST PS1 benzoic acid) and the successful organization of multi-laboratory round-robin trials have demonstrated that qNMR can achieve the precision and accuracy required for GMP environments [73,74,75]. These regulatory and metrological advances, combined with the technological frontiers discussed above, are lowering the barriers to routine qNMR deployment in quality control laboratories worldwide. Finally, the integration of all these technologies, AI-driven processing, QM-based interpretation, hyperpolarization-enhanced sensitivity, portable benchtop platforms, and regulatory harmonization, will synergistically lower the barriers to qNMR adoption. From automated quality control in pharmaceutical manufacturing to real-time metabolic monitoring in clinical settings, qNMR is destined to expand its role far beyond the specialized spectroscopy laboratory. By navigating these frontiers, qNMR will cement its position as an irreplaceable tool for discovery, development, and quality assurance in science and industry.

## Figures and Tables

**Table 1:** Reported internal calibrants for qNMR analysis.

Compounds	CAS Number	Advantages	Disadvantages
2,4,6-Triiodophenol	609-23-4	Singlet	Expensive; potential iodine-related artifacts
1,3,5-Trichloro-2-nitrobenzene	18708-70-8	Singlet; stable	Limited solubility; toxic
3,4,5-Trichloropyridine	33216-52-3	Singlet; stable	Toxic; limited solubility in water
Dimethyl terephthalate	120-61-6	Two singlets	May overlap with methoxy signals
1,4-Dinitrobenzene	100-25-4	Aromatic singlet	Potential π-π stacking with aromatic analytes
1,3,5-Triiodobenzoic acid	88-82-4	Aromatic singlet	Expensive; poor solubility in water
Maleic acid	110-16-7	Singlet; inexpensive; non-volatile	Acidic; may react with basic analytes
Fumaric acid	110-17-8	Singlet; stable; non-hygroscopic	Poor solubility in organic solvents
Sodium *p*-hydroxybenzoate	5026-62-0	Water-soluble; two doublets	Complex multiplet pattern
Dimethyl sulfone	67-71-0	Singlet; soluble in wide range of solvents	May overlap with methoxy or *N*-methyl signals
TSP-*d*_4_ (sodium salt)	5683-30-7	Singlet; water-soluble	Hygroscopic
Hexamethylphosphoramide	680-31-9	Sharp singlet	Carcinogenic; toxic; hygroscopic
Ethylene carbonate	96-49-1	Stable; non-hygroscopic	Limited solvent compatibility
1,4-Dioxane	123-91-1	Singlet	Volatile; can form peroxides
2-Ethylphenol	90-00-6	Inexpensive	Multiple signals
Benzoic acid	65-85-0	Stable; soluble in many organic solvents	Multiple signals; acidic
Potassium hydrogen phthalate	877-24-7	non-Hygroscopic; stable	Complex aromatic multiplet pattern
Ethylparaben	120-47-8	Inexpensive	Multiple signals
Acesulfame potassium	55589-62-3	Singlet; stable	Limited to aqueous solvents
1,3,5-Trimethoxybenzene	621-23-8	Two singlets; soluble in organic solvents	May overlap with methoxy signals
3,5-Dinitrobenzoic acid	99-34-3	Stable; low-field signals	Poor solubility in non-polar solvents; acidic
Methyl 3,5-dinitrobenzoate	2702-58-1	Stable; low-field signals	Less water-soluble

**Table 2:** Recommended acquisition and processing parameters for routine qNMR analysis.

Parameter	Recommended Setting	Notes/Rationale
Sample preparation		
Solvent selection	DMSO-*d*_6_, CDCl_3_, CD_3_OD, D_2_O	Choose based on analyte solubility and signal dispersion
Sample concentration	5–20 mg/mL for a 5 mm tube	Adjust to achieve S/N ≥ 150 for peaks of interest
Internal calibrant concentration	Approximately equimolar to analyte	Minimizes integration errors
Acquisition		
Temperature	298 K (controlled)	Maintain constant temperature for reproducibility
Flip angle	30° or optimized Ernst angle	Smaller angles allow shorter D1
Relaxation delay (D1)	5–7 × longest T1	For 90° pulse; determine T_1_ via inversion recovery
Number of scans (ns)	128–1024	Adjust to achieve the desired S/N for target peaks
Spectral width (SW)	20–30 ppm	Centered at ca. 5–10 ppm
Acquisition time (AQ)	2–4 s	Balances resolution and relaxation contribution
Shimming	Manual or gradient shim	Essential for symmetrical line shapes
^13^C decoupling (optional)	GARP decoupling during acquisition only	Collapses ^13^C satellites; reduces spectral crowding
Processing		
Line broadening (LB)	0.1–0.3 Hz	Or Gaussian multiplication for resolution enhancement
Zero filling	2–3× original data points	Typical final size: 128 K (400–500 MHz) or 256 K (≥600 MHz)
Phasing	Manual	More accurate than automatic routines
Baseline correction	Polynomial fit	Achieve a visually flat baseline at zero intensity

## Data Availability

No new data were created or analyzed in this study. Data sharing is not applicable to this article.
